# A CDC WONDER Study on Disparities in Place of Death Among Patients With Systemic Lupus Erythematosus in the United States between 1999 and 2020

**DOI:** 10.7759/cureus.89313

**Published:** 2025-08-04

**Authors:** Aruna Anantharaj, Dr.Rohit Rao Yelagapuri, Parth Vikram Singh, Aayza Nadeem, Shreya Deoghare

**Affiliations:** 1 Internal Medicine, Wuhan University School of Medicine, Hubei, CHN; 2 Intensive Care Unit, Ramdev Rao Hospital, Hyderabad, IND; 3 Internal Medicine, Indira Gandhi Government Medical College, Nagpur, IND; 4 Internal Medicine, Jinnah Sindh Medical University, Karachi, PAK; 5 Dermatology, N.K.P. Salve Institute of Medical Sciences and Research Centre, Nagpur, IND; 6 Dermatology, Alexis Multi-speciality Hospital, Nagpur, IND

**Keywords:** autoimmune, hospice care, mortality trends, neuropsychiatric systemic lupus erythematosus (npsle), systemic lupus erythematosus

## Abstract

Introduction: Systemic lupus erythematosus (SLE) is an autoimmune disease, more prevalent among African-American women, often associated with severe manifestations such as lupus nephritis and neuropsychiatric lupus. Both conditions contribute significantly to morbidity and mortality, though lupus nephritis is more commonly linked to direct disease-related deaths. Mortality can also result from other severe disease manifestations or treatment-related complications. Assessing disparities in place of death among lupus patients is essential for improving care quality through targeted physician and patient education.

Aims: The aim of this study is to evaluate disparities in the place of death (hospital, nursing home, hospice, and home) among lupus patients in the United States of America (USA), based on demographic characteristics like age, gender, race, and geographic regions.

Methodology: A retrospective study was conducted in September 2023 using Centers for Disease Control and Prevention - Wide-Ranging Online Data for Epidemiologic Research (CDC WONDER) data, focused on deaths attributed to lupus erythematosus (International Classification of Diseases, Tenth Revision (ICD-10) Code: L93, including L93.0, L93.1, and L93.2). It analyzed disparities in death location (home/hospice, medical facility/nursing home, others that include unknown locations or unspecified categories where the place of death could not be conclusively determined) across four variables employing statistical tools STATA15 (StataCorp LLC, College Station, TX) and R3.6.3 (The R Core Team, R Foundation for Statistical Computing, Vienna, Austria).

Results: A total of 3,937 deaths from 1999 to 2020 were included in the analysis from the CDC WONDER database, which showed 1,269 deaths were in home/hospice, 2,517 in medical/nursing facilities, and 151 at other unknown/unspecified locations. Higher odds of home/hospice deaths were noted in ages 55-64 years (1.606) and 65-74 years (1.576), males (1.306 times more than females), patients who were American Indian/Alaska Native (3.447), and those in Census region 3: South (1.459).

Conclusions: Lupus patients above 50 years, males, and those of American Indian/Alaskan race, and from the Census region 3 (South) have an increased likelihood of dying in home/hospice care. Future predictions till 2025 using the AutoRegressive Integrated Moving Average (ARIMA) model indicate an increasing trend of cumulative home/hospice mortality.

## Introduction

Systemic lupus erythematosus (SLE), or lupus, an autoimmune disorder characterized by aberrant immune system activation, can result in a wide spectrum of cutaneous and clinical symptoms [1]. The genetic and phenotypic heterogeneity of SLE contributes to its varying symptom presentation and disease progression, making it challenging to diagnose and treat [1]. Although prevalent in all genders and races, it is common among African American women [1]. Treatment of SLE typically includes corticosteroids, immunosuppressants such as methotrexate and mycophenolate mofetil, plasmapheresis, and biologic drugs [2]. Lupus nephritis, the most common target-organ manifestation, requires tailored treatment strategies to maximize efficacy while minimizing toxicity [1]. The common causes of mortality in lupus patients include lupus nephritis and neuropsychiatric lupus, along with secondary complications such as bacterial infections and cardiovascular diseases. Other causes include bacterial infections and cardiovascular complications [2]. An estimated 10% to 15% of lupus patients die prematurely due to complications of lupus. However, with improvements in diagnosis and treatment, most lupus patients will go on to live a normal life span [3].

A study by Pryor et al. identified a high prevalence of lupus and lupus nephritis among Medicaid recipients, signifying obstacles to receiving high-quality lupus care and drug adherence. This can lead to disparities in adverse outcomes and mortality due to lupus [4]. A study by Yen et al. reported that SLE ranked among the top 20 causes of death in females aged between five to 64 years. Additionally, among Black and Hispanic women, SLE was placed fifth in the 15-24 age group, sixth in the 25-34 age group, and eighth-ninth in the 35-44 age group [5].

The topic of death and dying has not received substantial attention in healthcare conversations between physicians and patients, mostly due to uneasiness around this topic [6]. Although not universal, death at home or in a hospice facility is considered preferable to death in an inpatient hospital or skilled nursing facility [6]. Although hospitals and nursing facilities can provide skilled treatment measures, hospice care adds to the quality of care and these patient-centered priorities, like being physically independent and having good symptom control [7]. With the purpose of physician and patient education and ensuring superior quality of care, it is necessary to evaluate trends and disparities in the place of death of patients suffering from SLE.

The primary aim of this study is to evaluate disparities in the place of death (hospital, nursing home, hospice, and home) among lupus patients in the USA. The secondary aim is to evaluate the disparities based on demographic characteristics like age, gender, race, and geographic region.

## Materials and methods

Data source

A cross-sectional observational study was conducted in September 2023, using an online, publicly available database, namely the Centers for Disease Control and Prevention Wide-Ranging Online Data for Epidemiological Research (CDC WONDER) [8].

Data collection

Data were extracted on 2^nd ^September 2023 from the CDC WONDER website. The option “Underlying Cause of Death by Bridged-Race Categories from the years 1999-2020” was selected. The cause of death was selected using International Classification of Diseases, Tenth Revision (ICD-10) codes. The code for lupus erythematosus was L93 [8].

Variables selected were as follows: age, gender, race (American Indian or Alaska Native, Asian or Pacific Islander, Black or African American, or White), US census regions (Northeast, Midwest, South, and West), and place of death (medical facility-inpatient, outpatient, or emergency room; dead on arrival; home; hospice facility; nursing home or long-term facility; unknown place of death; and others, including unknown location or unspecified categories). Places of death were combined into three main groups (home or hospice, medical facility or nursing home, and others).

Statistical analysis

The data were extracted from the CDC WONDER database and imported into Microsoft Excel (Microsoft Corp., Redmond, WA) for further analysis. All statistical analyses were conducted through statistical analysis tool STATA Version 15 (StataCorp LLC, College Station, TX) and R Version 3.6.3 (The R Core Team, R Foundation for Statistical Computing, Vienna, Austria) and included univariate logistic regression, with p-value < 0.05 considered statistically significant. 

## Results

A total of 3,937 deaths were reported in lupus patients as per data obtained from the CDC WONDER database from 1999 to 2020 in the United States. Out of this, 1,269 deaths were reported in home or hospice care, 2,517 in medical or nursing facilities, and 151 at other places.

Table 1 shows the place of death of lupus patients based on age, gender, census region, and race, in absolute numbers. When age groups are considered, deaths at home or in hospice were seen highest in the 55-64 age group (n=273, 21.5%), ); and deaths in medical or nursing facilities were highest for the 45-54 age group (n=416, 16.5%), followed by the 55-64 age group (n=411, 16.3%). The number of deaths was higher in medical or nursing facilities compared to at home or in hospice for all age groups, both genders, all four census regions, and three races, except for the American Indian or Alaska Native race, wherein 15 lupus patients (1.2%) died at home or in hospice compared to 13 (1.02%) in medical and nursing facilities.

**Table 1:** Place of death of lupus patients based on age, gender, census region, and race in absolute numbers Values have been reported as N values.

Variables	Home or hospice (n = 1269)	Medical facility or nursing (n = 2517)	Others (n = 151)
Ten-year age groups			
5-14 years	0	13	0
15-24 years	18	138	0
25-34 years	55	269	0
35-44 years	133	354	14
45-54 years	248	416	22
55-64 years	273	411	14
65-74 years	225	333	24
75-84 years	219	340	28
85+ years	92	209	21
Gender			
Female	1052	2171	133
Male	217	346	18
Census region			
Census region 1: Northeast	137	369	10
Census region 2: Midwest	250	560	26
Census region 3: South	671	1182	90
Census region 4: West	211	392	24
Race			
American Indian or Alaska Native	15	13	0
Asian or Pacific Islander	15	72	0
Black or African American	321	930	29
White	918	1489	106

Table 2 shows predictors of home or hospice deaths of lupus patients. Based on age groups, considering 85+ years of age as a reference, the odds of death at home or in hospice were highest for the 55-64-year-old age group (1.606 times, 95% CI (1.207, 2.137), p <0.05), followed by the 65-74-year-old age group (1.576 times, 95% CI (1.175, 2.114), p <0.05) and the 75-84-year-old age group (1.488 times, 95% CI (1.109, 1.996), p <0.05). The odds of a male having a home or hospice death were 1.306 times that of a female, with a 95% CI of 1.087, 1.568. The odds of death in home hospice were highest for American Indian or Alaska Native patients (3.447 times (95% CI: 1.623, 7.322)) compared to the reference.

**Table 2:** Predictors of home or hospice deaths of lupus patients Values have been reported as odds ratios and 95% confidence intervals. P-value <0.05 is significant.

Variables	Univariate logistic regression		
	Odds ratio	95% CI	P-value
Age			
5-14 years	0	(0, 2.956e+202)	0.956
15-24 years	0.326	(0.189, 0.564)	<0.001*
25-34 years	0.511	(0.35, 0.746)	<0.001*
35-44 years	0.904	(0.661, 1.235)	0.525
45-54 years	1.416	(1.062, 1.887)	0.018*
55-64 years	1.606	(1.207, 2.137)	0.001*
65-74 years	1.576	(1.175, 2.114)	0.002*
75-84 years	1.488	(1.109, 1.996)	0.008*
85+ years	1.0 (Reference)		
Gender			
Male	1.306	(1.087, 1.568)	0.004*
Female	1.0 (Reference)		
Census region			
Census region 1: Northeast	1.0 (Reference)		
Census region 2: Midwest	1.18	(0.924, 1.508)	0.185
Census region 3: South	1.459	(1.175, 1.812)	0.001*
Census region 4: West	1.403	(1.086, 1.813)	0.01*
Race			
American Indian or Alaska Native	3.447	(1.623, 7.322)	0.001*
Asian or Pacific Islander	0.622	(0.352, 1.101)	0.103
White	1.719	(1.48, 1.998)	<0.001*
Black or African American	1.0 (Reference)		

**Figure 1:**
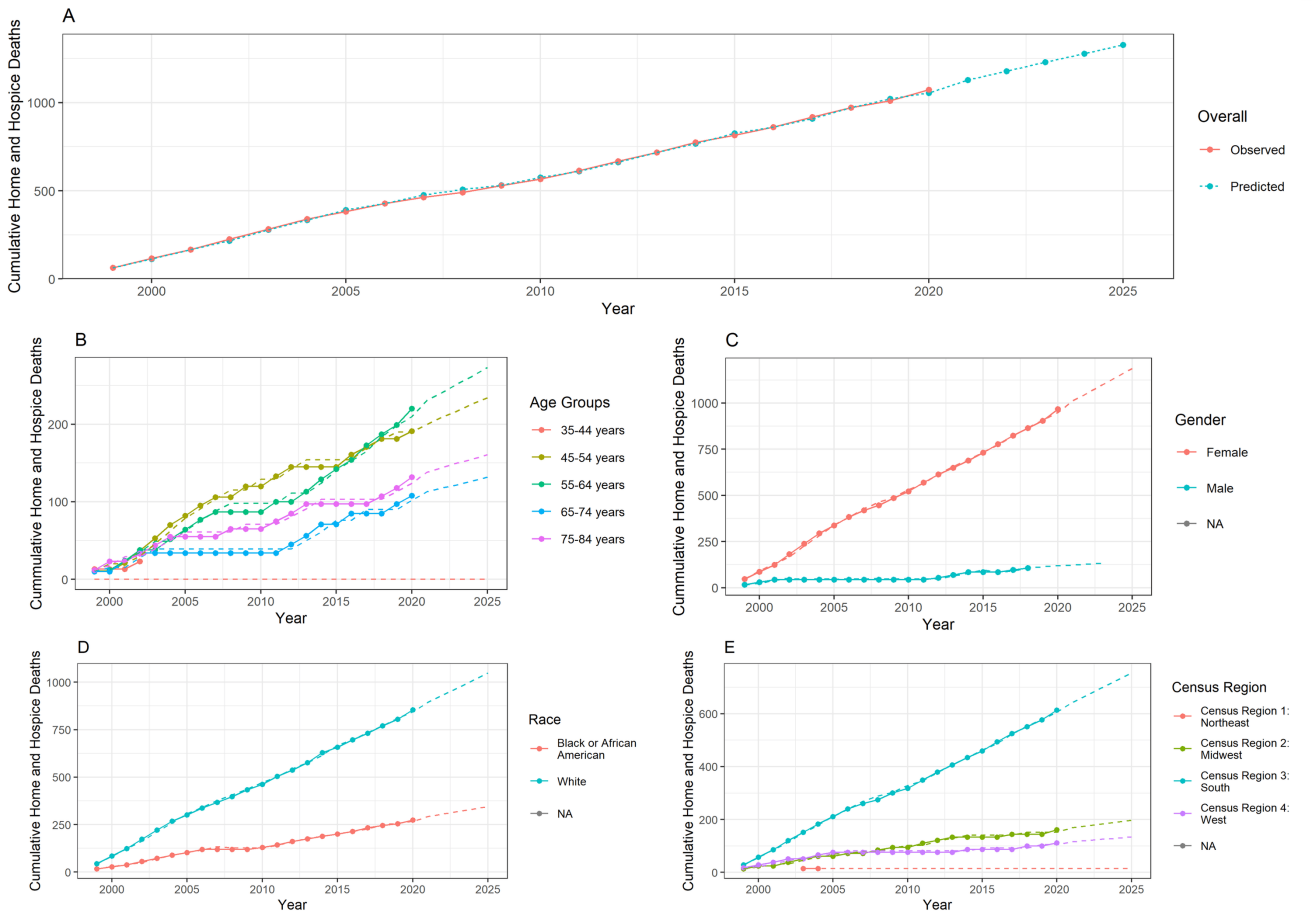
Cumulative home or hospice death trends A: Overall observed and predictive mortality trends; B: Mortality trends on the basis of 10-year age groups; C: Mortality trends on the basis of gender; D: Mortality trends on the basis of race; E: Mortality trends on the basis of census region NA: none of the above

Figure 1 shows the cumulative home or hospice death trends of lupus patients. The lines in the chart represent the observed data, which is available from the years 1999-2020, and the dotted line represents the prediction of deaths till 2025. The method used is the AutoRegressive Integrated Moving Average (ARIMA) model. The cumulative deaths in home and hospice are predicted to reach more than 1500 by the year 2025 (Figure 1A). The cumulative deaths in homes or hospices for age groups 55-64 years and 45-54 years are predicted to exceed 200 each by 2025 (Figure 1B). For females, the number is predicted to exceed 1,000 and remain fairly stable, while for males, it is predicted to remain around 200 (Figure 1C). The cumulative home and hospice deaths are predicted to increase rapidly for lupus patients of the White race (Figure 1D) and Census region 3: South (Figure 1E), compared to their counterparts of other races and census regions, respectively.

## Discussion

This retrospective study using the CDC WONDER database from 1999 to 2020 revealed that 3,937 deaths (1,269 at home or in hospice, 2,517 in medical or nursing facilities, and 151 at other places) were reported in lupus patients. The odds of death at home or in hospice were highest for age groups 55-64 years (1.606) and 65-74 years (1.576) compared to the reference; male gender (1.306 times more than female); American Indian or Alaska race (3.447) compared to the reference; and Census region 3: South (1.459) compared to the reference. The cumulative home or hospice death trends of lupus patients seem to be rising and are predicted to cross 1,500 by the year 2025. The rise in mortality is mainly for ages 55-64 years and 45-54 years, females, the White race, and Census region 3: South, compared to their respective counterparts.

In our study, we found that participants between 15-24 and 25-34 years exhibited significantly reduced odds of dying at home or in hospice care, with odds ratios (ORs) of 0.326 and 0.511, respectively (P-value <0.001). Interestingly, we observed an incremental increase in the ORs in older age groups, peaking in the 55-74 years age range, consistent with Figure 1B, which visualizes this 10-year age group trend. These findings echo prior research highlighting the influence of age on healthcare utilization and decision-making at the end of life [9, 10].

Lupus is highly prevalent in females [1, 11]. As a result, our study demonstrated a higher absolute number of deaths across all settings compared to their male counterparts. While the gender dynamics revealed compelling insights into lupus mortality, it's equally important to consider the intrinsic characteristics of SLE in influencing these trends. SLE is a long-term illness causing chronic inflammation, flare-ups of disease, persistent drug-induced toxicity, recurrent admissions, and prolonged hospital stays. This may explain why most deaths occur in medical facilities or nursing homes, underscoring the disease's severity and the consequent need for medical care [12]. Males had shown higher odds (OR 1.306, P-value = 0.004) of dying at home or in hospice care than females. Given the substantial number of female deaths in medical or nursing facilities, our study emphasizes the need for targeted interventions focusing on the female population [13].

The stark racial and ethnic variation is clearly visible from our data pool. American Indian or Alaska Native patients exhibited a considerably higher propensity for home or hospice deaths (OR 3.447, P-value = 0.001), while Asian or Pacific Islander patients had decreased odds (OR 0.622, P-value = 0.103). Notably, White American patients showed significantly increased odds (OR 1.719, P-value <0.001), underscoring the existing racial disparities in healthcare accessibility and decision-making. Our findings delineate the racial and ethnic variations in end-of-life care choices, emphasizing the persistent racial disparities in healthcare accessibility and decision-making, in alignment with conclusions drawn in an earlier study [14, 15].

Regionally, the South census region displayed higher mortality in all settings, corroborated by Figure 1E. With an odds ratio of 1.459 (P-value = 0.001), the South has significant differences compared to the Northeast (reference region). The West also showed an elevated inclination towards home or hospice deaths (OR 1.403, P-value = 0.01). This regional variation could be attributed to localized healthcare policies and cultural factors affecting end-of-life care choices. Examining the healthcare coverage landscape and accessibility to medical services across regions can offer additional insights into observed regional disparities.

Understanding where patients with lupus are most likely to die is valuable for healthcare providers in emergency medicine and palliative care settings. Our study found that a substantial proportion of patients with lupus die in medical facilities, thereby highlighting the crucial need for end-of-life care discussions to be woven into routine clinical practice [16, 17]. Furthermore, since the likelihood of home or hospice death increases significantly in older age groups and varies by gender and region, these insights could serve as catalysts for developing targeted interventions aimed at enhancing end-of-life care planning, tailored to the unique needs of individual patients and their families [18].

Our forecasting model (Figure 1A) projects a concerning increase in mortality rates from lupus for the next five years. Utilizing the ARIMA model, the forecast aligns with observed data up to 2020 and suggests a trend that warrants immediate attention from healthcare systems and policymakers [19] to reassess and strengthen the palliative and emergency care protocols.

Our study had some limitations. Firstly, the focus on lupus-specific mortality without considering comorbidities, which might affect the place of death. Secondly, the data is limited to the year 2020, missing recent advancements and potential shifts in healthcare practices affecting lupus-related mortality from 2021 to 2023. Thirdly, we do not differentiate between lupus subtypes like SLE and cutaneous lupus erythematosus, which can have distinct clinical courses and complications, which potentially limits applicability to specific patient subgroups and masks variations in mortality and place of death that could be crucial for individualized patient care. Finally, smaller sample sizes for some age groups warrant cautious interpretation of these particular trends.

For future directions, it would be beneficial to explore the psychosocial as well as economic factors that influence end-of-life choices among lupus patients. Studies examining the characteristics and policies of healthcare systems may also offer additional insights. Potential areas for future research could include understanding caregiver roles in end-of-life choices [20, 21] and the impact of healthcare accessibility and affordability on these decisions. Such comprehensive inquiries would enable a nuanced approach to improving end-of-life care for this patient population [22].

## Conclusions

In conclusion, our study highlights significant disparities in the place of death among lupus patients, based on age, gender, race, and census region. Higher odds of home or hospice deaths were observed in individuals over 50, males, and those of American Indian or Alaska race in Census region 3: South. Predictive analysis reveals a concerning rise in such deaths among White emale lupus patients at home or hospice centers by 2025.

These findings underscore the importance of tailoring support systems for specific populations and prompt a call to action for healthcare professionals. By recognizing and addressing these disparities in mortality based on age, gender, and race, we can strive towards a healthcare system that provides personalized and culturally competent end-of-life care for lupus patients, advocating for a more compassionate and inclusive approach in lupus care.
